# The Role of Parental Influences in Chinese Adolescents’ Academic Achievement through Shaping Friendship Network Dynamics

**DOI:** 10.1007/s10964-025-02306-5

**Published:** 2026-01-05

**Authors:** Xingna Qin, Simeng Li, Björn Sjögren, Ping Ren, Robert Thornberg

**Affiliations:** 1 Department of Behavioral Sciences and Learning, Linköping University, Linköping, Sweden; 2 Collaborative Innovation Center of Assessment toward Basic Education Quality, Beijing Normal University, Beijing, China

**Keywords:** Friendship selection and influence, Academic achievement, Parental autonomy support and control, Parental expectations, Social network analysis

## Abstract

Parents and friends are critical social influences on adolescents’ academic achievement, yet how they function together within dynamic friendship networks remains insufficiently understood. This study examined how parental influences (autonomy support, behavioral control, psychological control, and educational expectations) interact with friendship selection and influence processes to shape academic achievement of Chinese adolescents. A total of 1,419 Chinese adolescents (*M*_*age*_ = 12.35, *SD* = 3.04; 46.4% girls) participated in four-wave surveys across two years. Longitudinal social network analyses revealed a clear friendship influence process, with adolescents’ achievement becoming increasingly similar to that of their friends over time. Parental influences operated through distinct pathways: perceptions of high parental expectations directly increased achievement, whereas autonomy support and psychological and behavioral control had no direct effect. Adolescents perceiving high parental autonomy support were more likely to select high-achieving friends; other parental influences did not affect friendship selection. No parental influences moderated the effect of friends influence on achievement. Overall, these findings suggest that parents shape adolescents’ achievement by setting expectations and providing support, either directly or via friendship selection, while friends exert an independent influence on achievement. Taken together, the results highlight how both parents and friends contribute adolescents’ academic development in the Chinese context.

**Supplementary Information:**

The online version contains supplementary material available at 10.1007/s10964-025-02306-5.

## Introduction

Academic achievement during adolescence is a key predictor of future success (Crosnoe & Benner, 2015). As adolescents seek greater independence and peer status, they become increasingly susceptible to the influence of their friends (Sawyer et al., 2012). Friendship can shape adolescents’ behaviors, attitudes, and academic outcomes through both selection and influence processes (Gremmen et al., 2017; Wentzel, 2017). At the same time, parents continue to impact adolescents’ achievement through their practices, such as providing support and exercising control (Vasquez et al., 2016), as well as through their expectations (Pinquart & Ebeling, 2020). Although the effects of friendships and parental influences have been extensively studied separately, their interactive role in shaping academic achievement, particularly within friendship networks, remains less understood. Recent social network research offers valuable insights into how parental influences may affect friendship selection related to achievement (Qin et al., 2024), yet it is still unclear how multiple domains of parental influence interact with adolescent friendship networks to shape the co-evolution of friendships and academic achievement. Therefore, this study uses longitudinal social network analysis to examine how parental influences, including autonomy support, behavioral control, psychological control, and educational expectations, interact with friendship selection and influence processes to shape the academic achievement of Chinese adolescents.

### Friendship Selection and Influence in Academic Achievement

Friendship plays a critical role in shaping adolescents’ academic achievement, particularly through the selection and influence process. According to the Wisconsin model of status attainment, significant others, including friends, influence an individual’s achievement by communicating achievement expectations, defining educational norms, and providing role models (Sewell et al., 1969). During adolescence, peers become increasingly salient as adolescents strive for peer acceptance and social belonging (Brechwald & Prinstein, 2011). The influence-compatibility model posits that friendship influence enhances peer similarity over time, as adolescents adapt their behaviors, attitudes, and academic goals to align with those of their friends (Laursen & Veenstra, 2021). This process is further supported by Social Learning, which holds that individuals learn behaviors through the observation and imitation of valued peers Theory (Bandura, 1977). High-achieving friendships can enhance academic performance by providing emotional support, sharing learning strategies, and reinforcing the value of education (Wentzel, 2017). Conversely, friendships with low-achieving peers can exert downward influences, leading to diminished academic performance (Gremmen et al., 2017). Notably, studies have found that the downward influence from low-achieving friends is stronger than the upward influence from high-achieving peers, both among German (Stark et al., 2017) and Chinese (Qin et al., 2024) adolescents, highlighting the asymmetric nature of peer influence.

Beyond influence effects, friendship selection represents another essential process through which adolescents’ academic outcomes are shaped. The upward or downward influence of friendships on achievement largely depends on the types of friends adolescents choose. Adolescents tend to select friends whose academic achievements are similar to their own (Gremmen et al., 2017; Laninga-Wijnen et al., 2019), which is known as “homophily”. This preference for academically similar peers is driven by shared interests, mutual understanding, and the desire for accurate self-evaluation through social comparison (Festinger, 1954). Empirical studies have consistently shown that high-achieving students are more likely to befriend other high-achievers, while low-achieving students tend to form friendships with peers of similar academic standing (Gremmen et al., 2017; Rambaran et al., 2017). Additionally, high-achieving and low-achieving students often avoid forming friendships with one another (Gremmen et al., 2017). Social exchange theory offers an explanation for this phenomenon, suggesting that high-achieving students may perceive low-achieving ones as lacking the academic resources or support necessary for mutually beneficial relationships (Homans, 1958). However, evidence suggests that this pattern is more nuanced. Among Chinese adolescents, for instance, some middle-achieving students actively seek friendships with high-achieving peers or those whose achievements exceed their own—a mechanism known as attractiveness (Qin et al., 2024). This behavior is often motivated by a desire to improve academic standing and is particularly encouraged in cultures where academic success is highly valued. Thus, while selection processes generally favor “homophily”, contextual factors such as cultural emphasis on academic achievement can lead to exceptions where students strategically form friendships to enhance their educational outcomes.

### Parental Influences, Achievement, and Adolescents Friendship

Parents can influence adolescents’ academic achievement both directly through socialization and indirectly through the management of peer contexts. According to the conceptual framework proposed by Ladd and Pattit (2002), parents shape their children’s peer relationships by guiding, supervising, and structuring their social opportunities. This process operates through two key mechanisms. First, parents shape adolescents’ friendship selection. Parental influences, such as support, monitoring, discipline, knowledge, and direction regarding friendships have been found to encourage adolescents to affiliate with prosocial or high-achieving peers (e.g., Mounts, 2008; Qin et al., 2024) and discourage affiliation peers engaged in deviant or risky behaviors, such as smoking and alcohol use (Hoeben et al., 2021; Lakon et al., 2015; Tilton-Weaver et al., 2013). Second, parents can moderate the influence of friends on adolescents’ outcomes. For instance, communicating disapproval can buffer against negative peer influences, reducing the impact from delinquent friends (Tilton-Weaver et al., 2013), whereas other dimensions, such as parental discipline or knowledge, may amplify the influence of friends’ deviant behaviors, such as alcohol use (Hoeben et al., 2021).

However, previous research has mostly focused on negative behaviors and paid less attention to how these parental influences shape friendship dynamics in relation to academic achievement. Moreover, parental influences are multifaceted; for instance, directing and monitoring behaviors may include both supportive and intrusive components (Qin et al., 2024). This makes it necessary to examine the supportive and controlling dimensions separately. Guided by Self-Determination Theory, the present study focuses on three core parenting practices —autonomy support, behavioral control, and psychological control— defined by their capacity to support or thwart adolescents’ basic psychological needs (Ryan & Deci, 2017), as well as on a key parental value: educational expectations. This focus is particularly relevant in the Chinese context, where parents typically hold very high educational expectations of their children. This is embodied in the idiom “*wang zi cheng long*” (望子成龙, meaning “hoping one’s son will become a dragon”), which reflects the widespread belief that parents bear a duty to ensure their children’s educational success (Cheah et al., 2019). When adolescents internalize these high expectations and align their personal ambitions with filial duty, they may become strongly motivated to perform well academically (Liew et al., 2014).

### Parental Autonomy Support, Adolescents’ Friendship Selection and Influence, and Achievement

Parents remain a vital source of social support for adolescents, even as youth become increasingly independent and peer-oriented (Sawyer et al., 2012). One key form of support is *parental autonomy support*, characterized by encouraging children’s initiative and acknowledging their perspectives. Such support enhances positive school engagement, fostering intrinsic motivation and positive academic outcomes (Pomerantz et al., 2007). As adolescents achieve higher levels of academic success, they are subsequently more likely to affiliate with high-achieving peers. This initial selection process is primarily driven by the mechanism of “homophily”—the tendency for individuals to associate with others who are similar to themselves.

Beyond this direct academic pathway, autonomy-supportive parenting also fosters internal assets that can shape adolescents’ friendship selection. According to Self-Determination Theory (Ryan & Deci, 2017), autonomy-supportive parenting nurtures adolescents’ basic psychological needs for autonomy, competence, and relatedness, thereby promoting self-regulation, social competence, and intrinsic motivation. These internal assets are core drives for forming and maintaining high-quality friendships (Rubin et al., 2004). Consistent with Social Learning Theory (Bandura, 1977) and Attachment Theory (Bowlby, 1982), adolescents also learn and model supportive behaviors and emotional security from their parents, which in turn are reflected in the quality of their peer relationships. Through behaviors such as explaining rules, acknowledging emotions, and respecting friendship choices, autonomy-supportive parents indirectly equip adolescents with the social and emotional skills needed to build constructive, achievement-oriented peer relationships (McCurdy et al., 2020; Soenens et al., 2007). Consequently, adolescents with autonomy-supportive parents tend to affiliate with high-achieving, prosocial peers and are less likely to associate with deviant or low-achieving peers (Soenens et al., 2009).

Furthermore, autonomy-supportive parenting not only guides peer selection but also amplifies the positive influence of those peers. By fostering internal assets such as self-regulation and intrinsic motivation, parents enable adolescents to engage with their peers’ academic behaviors in a self-directed manner. Adolescents thus become more likely to internalize the positive academic attitudes of high-achieving peers, thereby reinforcing peer influence on academic success. Empirical evidence supports this interaction: the association between friends’ and adolescents’ school grades has been found to be stronger among those with authoritative parents (Mounts & Steinberg, 1995). Other forms of parental support exhibit similar buffering or enhancing effects. For example, parental affective support can protect adolescents from the negative influence of disengaged peers (Marion et al., 2014), whereas low parental warmth has been linked to stronger negative effects of low-achieving peers on educational aspirations (Espinoza et al., 2014). Overall, autonomy-supportive parenting fosters adolescents’ social and academic competencies, guides them towards selecting academically oriented friends, and strengthens the positive influence of these friendships on academic achievement.

### Parental Psychological and Behavioral Control, Adolescents’ Friendship Selection and Influence, and Achievement

Parental control, encompassing both psychological and behavioral dimensions, plays a significant role in adolescents’ academic achievement and friendship dynamics. *Psychological control*, which includes guilt induction and love withdrawal, undermines adolescents’ autonomy and self-worth, increasing the risk of maladaptive outcomes such as poorer academic performance and heightened psychological distress (Aunola & Nurmi, 2004). Conversely, *Behavioral control* refers to parents’ monitoring of their children’s activities, behaviors, and friendships. When applied appropriately, behavioral control is associated with stronger academic performance and lower levels of delinquency (Pinquart, 2016).

These distinct forms of parental control exert powerful yet opposing influences on adolescents’ friendship selection. According to Social Control Theory (Hirschi, 1969), parents exert direct influence over their children’s socialization contexts by reinforcing conformity to family norms and discouraging associations with deviant peers. Psychological control operates through negative pathways: by undermining social competence and self-worth, it increases the risk of peer rejection (Anaçali, 2023) and the likelihood of affiliating with deviant or low-achieving peers (Oudekerk et al., 2015; Tian et al., 2019). Behavioral control, conversely, guides peer selection through more constructive pathways. By promoting self-regulation and clarifying expectations, effective parental monitoring reduces affiliations with delinquent peers (Tilton-Weaver et al., 2013) and increases the likelihood of selecting academically oriented friends (Brown et al., 1993; Mounts, 2001).

Beyond shaping friendship selection, parental control also moderates the *influence* of friendships on achievement. Although direct research regarding its capacity to amplify *positive* peer influence is limited, evidence highlights its mitigating effects on negative peer influence. For instance, excessive psychological control can heighten adolescents’ susceptibility to deviant peer influence by fostering dependency and undermining internal motivation (Keijsers et al., 2012). In contrast, appropriate behavioral control— such as communicating disapproval of deviant behavior —can serve as a protective buffer against such delinquent peer influences (Tilton-Weaver et al., 2013). It is also plausible that self-regulation fostered through effective behavioral control enhances adolescents’ capacity to internalize and benefit from the positive academic behaviors of high-achieving peers.

### Parental Educational Expectations, Adolescents’ Friendship Selection and Influence, and Achievement

Parents can also influence adolescents’ academic achievement and friendship dynamics through their *educational expectations*. According to Expectancy–Value Theory (Wigfield & Eccles, 2002), parents’ academic expectations and the value they place on education play a significantly role in shaping adolescents’ motivation, beliefs, and achievement-related behaviors. When parents hold high expectations, adolescents tend to internalize these beliefs, which in turn predict greater academic achievement and higher educational attainment (Pinquart & Ebeling, 2020; Zhang et al., 2011).

These internalized values and goals directly affect adolescents’ friendship selection. Youth who have adopted high academic standards from their parents are naturally drawn to peers who share similar achievement orientations, a process consistent with the principle of homophily. Furthermore, parents often actively facilitate this alignment. In line with Social Control Theory (Hirschi, 1969), parental expectations reinforce adolescents’ commitment to conventional and achievement-oriented goals by shaping their children’s social environment. Parents may do so by encouraging participation in structured academic contexts—such as honor societies, tutoring groups, and extracurricular clubs—or by explicitly endorsing friendships with peers who demonstrate academic diligence (Zhu, 2020). This interaction of internalized values and external structuring channels adolescents toward academically oriented peer groups and away from deviant or disengaged social contexts.

Beyond shaping friendship selection, high parental expectations may also moderate the effects of friendship influence on academic achievement. Although direct empirical evidence remains limited, it is plausible that high parental expectations function as a stabilizing filter on peer influence. Adolescents raised in families where academic success is a core value tend to internalize a robust academic identity. This deeply ingrained identity may both enhance susceptibility to the positive academic behaviors of high-achieving friends (e.g., adopting effective study habits) and buffer against negative peer influences, such as peer groups who downplay the importance of school achievement.

## Current Study

Previous studies have mainly focused on friendship processes related to academic achievement separately, with limited attention to the simultaneous effects of friendship and parental influence. Furthermore, whether distinct parental factors shape achievement through interactions with friendship process remains underexamined in Chinese adolescents. To address these research gaps, this study employed longitudinal social network analysis to examine how parents and friends shape Chinese adolescents’ academic achievement, focusing on how specific parental influences (autonomy support, behavioral control, psychological control, and educational expectations) interact with dynamic processes of friendship selection and influence. Drawing on the relevant theories and prior research, it was hypothesized that adolescents’ academic achievement would be influenced by their friends’ achievement, becoming increasingly similar over time (Hypothesis1). Parental autonomy support (Hypothesis 2a), behavioral control (Hypothesis 2b), and expectations (Hypothesis 2c) were expected to positively predict academic achievement, whereas psychological control (Hypothesis 2d) was hypothesized to predict it negatively. In addition, adolescents perceiving higher parental autonomy support (Hypothesis 3a), behavioral control (Hypothesis 3b), and expectations (Hypothesis 3c) were hypothesized to be more likely to select high-achieving friends, while higher psychological control (Hypothesis 3d) was expected to reduce this likelihood. Finally, higher levels of parental autonomy support (Hypothesis 4a), behavioral control (Hypothesis 4b), expectations (Hypothesis 4c), and psychological control (Hypothesis 4d) were hypothesized to moderate friendship influence processes by strengthening the impact of these friendships on academic achievement.

## Method

### Participants and Procedure

The sample consisted of seventh-grade students from 26 classrooms across five mainland Chinese public middle schools. Data collected at four time points, each six months apart. Wave 1 (T1) occurred in the spring of seventh grade, Wave 2 (T2) in the fall of eighth grade, Wave 3 (T3) in the spring of eighth grade, and Wave 4 (T4) in the fall of ninth grade. About 1,419 students (46.4% girls, *M*_age_ = 12.35 years, *SD* = 3.04) participated in T1, 1,397 students (47.1% girls; *M*_age_ = 12.75 years, *SD* = 3.09) in T2, 1,363 students (47.8% girls; *M*_age_ = 13.27 years, *SD* = 3.19) in T3, and 1,300 students (50.8% girls; *M*_age_ = 13.70 years, *SD* = 3.27) in T4. Descriptive information about the sample characteristics, friendship networks, and classroom details is presented in Appendix 1 (Tables S1 and S2).

The study was approved by the Human Ethics Review Board of the Beijing Normal University. Students completed the questionnaire in classrooms during regular lessons, supervised by trained undergraduate or postgraduate students alongside the classroom teacher. Prior to participation, informed consent was obtained from parents or legal caregivers, and students volunteered to participate with their informed assent required at each assessment wave.

### Measures

#### Friendship (T1-T4)

 Friendship networks were constructed from peer nominations. Students nominated up to five “best friends” from a randomized class list, excluding self-nominations. These data were formatted into classroom-specific adjacency matrices for each wave, where ties were coded as present (1) or absent (0) a tie between two actors.

#### Academic achievement (T1-T4)

 Academic achievement was assessed using official school records from seven core subjects: Chinese, English, Mathematics, biology, history, geography, and politics. Scores were derived from standardized examinations conducted by all the schools at the end of every semester. A composite score was created from the seven subjects and Cronbach’s alpha for the seven-subject composite was 0.92 at T1, 0.90 at T2, 0.91 at T3 and 0.93 at T4. The total score was transferred into 9 categories, with 50 as an interval (< 250 to 1, 250 < x < 300 to 2, 300 < x < 350 to 3, 350 < x < 400 to 4, 400 < x < 450 to 5, 450 < x < 500 to 6, 500 < x < 550 to 7, 550 < x < 600 to 8, x > 600 to 9).

#### Parental autonomy support (T1)

 Parental autonomy support was measured by 8-item scale from Wang et al. (2007), including choice making (e.g., “My parents allow me to decide things for myself.”) and opinion exchange (e.g., “My parents trust me to do what they expect without checking up on me.”). Adolescents indicated how often each item occurred, with responses ranging from 1 (*never*) to 5 (*very often*). A mean score was computed, with higher scores indicating greater perceived parental autonomy support (Cronbach’s *α* = 0.88).

#### Parental psychological control (T1)

 Parental psychological control was measured using an 18-item scale reported by adolescents (Wang et al., 2007). The scale has three subscales, including *guilt induction* (e.g., “My parents tell me that I should feel ashamed when I do not behave as they wish.”), *love withdrawal* (e.g., “My parents avoid looking at me when I have disappointed them”), *authority assertion* (e.g., “My parents tell me that I am not as good as other kids when I fall short of their expectations”). Responses were captured on a 5-point frequency scale (1 = *never* to 5 = *very often*). A mean score was calculated, where higher values reflect greater perceived parental psychological control (Cronbach’s *α* = 0.90).

#### Parental behavior control (T1)

 Parental behavior control was assessed using a 16-item scale from Wang et al. (2007), including *parental restrictiveness* (e.g., “How often do your parents require you to speak with them before I decide on plans for weekends with my friends?”), *solicitatio*n (e.g., “My parents ask me about places I go with my friends?”). Adolescents reported the frequency of each behavior on a 5-point scale (1 = *never* to 5 = *very often*). A mean score was computed, with higher scores indicating greater perceived parental behavioral control (Cronbach’s *α* = 0.92).

#### Parental educational expectations (T1)

 At T1, parental educational expectations were assessed with single item, “What is your parents’ expectations for your highest level of education?”. Responses were recorded on a 6-point scale: 1 (graduate from middle school), 2 (graduate from high/vocational/technical school), 3 (graduate from high school), 4 (a vocational college degree), 5 (undergraduate degree), and 6 (a master’s degree or higher).

#### Demographic information (T1)

 Participants reported their gender and subjective socioeconomic status (SES) at T1. SES was measured using a five-point scale based on the question: “How would you rank your family’s financial situation?”. Rating ranged from 1 to 5, with higher values indicating a better financial situation.

### Analytical Strategy

#### RSiena

 Longitudinal social network analyses, named stochastic actor-oriented models (SAOMs), were used to analyze the co-evolution of friendship networks and behaviors. It reveals the inter-temporal change in academic achievement and friendship network over time and determines selection and influence effects (Ripley et al., 2024). The model is implemented in RSiena (Simulation Investigation for Empirical Network Analysis software package in R). The program enables the simultaneous estimation of friendship selection and influence processes regarding academic achievement, while controlling for structural network effects and the overall development of academic achievement in the network.

This study controlled several network structural effects based on previous studies (e.g., Qin et al., 2024) and the model fitting statistics. These included outdegree (density), reciprocity, transitive reciprocated triplets, transitive triplets, outdegree popularity, and outdegree activity. Behavior and friendship rate functions were also controlled to account for changes in academic achievement and network ties, respectively. The quadratic shape effect for achievement was included to model the tendency toward higher or lower achievement scores over time (Ripley et al., 2024). The explanations of some basic parameters in this RSiena model were provided in Table S3 in the Appendix 2.

Additionally, the proportion of missing data ranged from 6.3% to 12.8% for the friendship network, from 7.3% to 10.9% for academic achievement, and was 7.8% for parental factors. Missing data up to 20% are acceptable for network and covariate variables in the SAOM framework. RSiena handles missing data using “last observation carry forward” method (internal imputation procedure) that reduce their influence on parameter estimation (Huisman & Steglich, 2008).

#### Model specification for friendship influence processes

 The *average alter* effect was specified to test whether adolescents’ achievement become similar to their friends over time (H1). This parameter estimates whether adolescents with higher-achieving friends tend toward high academic achievement themselves, and conversely, whether adolescents with lower-achieving friends tend toward low achievement. When the *average alter* effect was significant, further *ego-alter* influence tables were calculated to examine the directional influence from friends, that is, whether friends influenced adolescents to increase or decrease in achievement over time (Ripley et al., 2024). Next, *effect from parameter* was used to test whether parental factors directly influence adolescents’ academic achievement over time (H2). Furthermore, the model controlled the effects of gender and subjective SES.

#### Model specification for friendship selection

 Friendship selection effects relating to achievement were used. Particularly, whether high-achieving adolescents were more likely to give (*ego effect*) and receive (*alter effect*) friendship nominations. Furthermore, *ego × alter* effect was used to detect whether adolescents form friendships based on similarity in academic achievement. Additionally, friendship selection effects relating to gender and subjective SES were also included as controlling effects.

#### Model specifications for moderate friendship selection and influence processes

 For friendship selection, *parental practices ego × achievement alter was used* to examine whether parental practices would enhance or reduce the likability of selecting high-achieving peers as friends (H3). For friendship influence, the *average alter × Effect from parental practices was used* to examine whether parental practices would enhance or reduce friendship influence regarding to academic achievement (H4). When the moderation effects were significant, interaction effects were further interpreted using further ego-alter selection tables for friendship selection (H3) and ego-alter influence tables for friendship influence (H4), following Ripley et al. (2024).

#### Meta-analytic procedure

 A meta-analysis was conducted to aggregate results across classrooms using the *metafor* package in R (Viechtbauer, 2010). To ensure reliability, only networks that successfully converged were included. Convergence required an overall maximum convergence ratio below 0.25 and *t*-ratios for convergence below 0.1 in absolute value for all parameters (Ripley et al., 2024). Based on these criteria, the final sample comprised 20 classrooms.

#### Goodness of Fit

 To assess whether the selected model accurately represented the observed data, a goodness-of-fit analysis was conducted for each class (Snijders & Steglich, 2015). A non-significant *p*-value indicates that the estimated model provides a good fit for the data. The results indicated a good fit for the indegree of friendship networks across all classrooms, with *p*-values ranging from 0.08 to 0.90.

## Results

### Descriptive Statistics

Table 1 reports descriptive statistics and correlations for the study variables. Academic achievement at T1 and T4 correlated positively with parental expectations (*r* from 0.40 to 0.43), parental autonomy support (*r* from 0.16 to 0.18), parental behavioral control (*r* from 0.11 to 0.15), and negatively with parental psychological control (*r* from –0.15 to –0.18).

**Table 1:** Descriptive results and correlations between academic achievement and parental influences

	1	2	3	4	5	6	7	8	9	10
1. SubSES T1	1									
2. Gender T1	0.02	1								
3. Parental expectations T1	–0.04	0.11^**^	1							
4. Parental psychological control T1	0.08	–0.12^**^	–0.08^**^	1						
5. Parental behavioral control T1	0.02	0.15^**^	0.13^**^	0.11^**^	1					
6. Parental autonomy support T1	–0.09^**^	0.06^*^	0.13^**^	–0.23^**^	0.28^**^	1				
7. Achievement T1	0.15^**^	0.22^**^	0.43^**^	–0.17^**^	0.15^**^	0.16^**^	1			
8. Achievement T2	0.12^**^	0.24^**^	0.42^**^	–0.15^**^	0.12^**^	0.18^**^	0.94^**^	1		
9. Achievement T3	0.12^**^	0.22^**^	0.40^**^	–0.18^**^	0.11^**^	0.17^**^	0.90^**^	0.91^**^	1	
10. Achievement T4	0.12^**^	0.25^**^	0.41^**^	–0.17^**^	0.14^**^	0.16^**^	0.90^**^	0.91^**^	0.93^**^	1
Mean	3.30	0.47	5.07	2.78	3.57	3.44	5.50	5.38	6.39	5.89
*SD*	–	–	1.12	0.77	0.91	0.87	2.19	2.21	2.38	2.45

^*^ *p* < .05, ^**^p < .01

Table 2 presents the descriptions of the sample, friendship networks, and changes in networks and achievement. The average number of friendship nominations given varied between 3.35 and 3.98 across the four waves. The friendship networks exhibited a moderate level of reciprocity, with approximately 50%–53% of friendship nominations being reciprocated. There was also a tendency for friendships to occur in cohesive subgroups, indicated by a transitivity index in the network of from 31% to 36%. Further, same-gender friendships predominated, accounting for roughly 94%–97% of friendship nominations across waves. Moran’s I ranged from 0.19 to.26 for academic achievement across the four waves, which indicates a positive correlation between friends’ academic achievement. The Jaccard index values in three periods were 0.34, 0.35 and 0.36 in this study, which exceeds the threshold of 0.3 and indicates sufficient change and stability in the friendship networks (Veenstra & Steglich, 2012).

**Table 2:** Description of the sample and changes in friendship networks

	Wave 1	Wave 2	Wave 3	Wave 4		Wave1-Wave2	Wave2-Wave3	Wave3-Wave4
Average degree	4.08	3.82	3.43	3.24	Friendship change			
Density	0.07	0.07	0.06	0.06	Hamming distance	211	182	169
Reciprocity	0.53	0.50	0.51	0.53	Jaccard index	0.34	0.35	0.36
Transitivity	0.31	0.32	0.34	0.36	Friendship tie change			
Same gender	0.97	0.95	0.95	0.94	Ties dissolved	3097	2887	2398
Missing fraction	0.01	0.01	0.02	0.02	Ties emerged	2721	2399	2131
Moran’s I Achievement	0.20	0.19	0.22	0.26	Ties maintained	2847	2579	2493

The Hamming distance reflects the total number of nominations in the network. Jaccard index is the fraction of stable ties relative to all new, lost, and stable ties. Moran’s I network autocorrelation coefficient indicates the degree to which friends display similarity in terms of achievement

### Friendship Selection and Influences in Academic Achievement

**Fig. 1:**
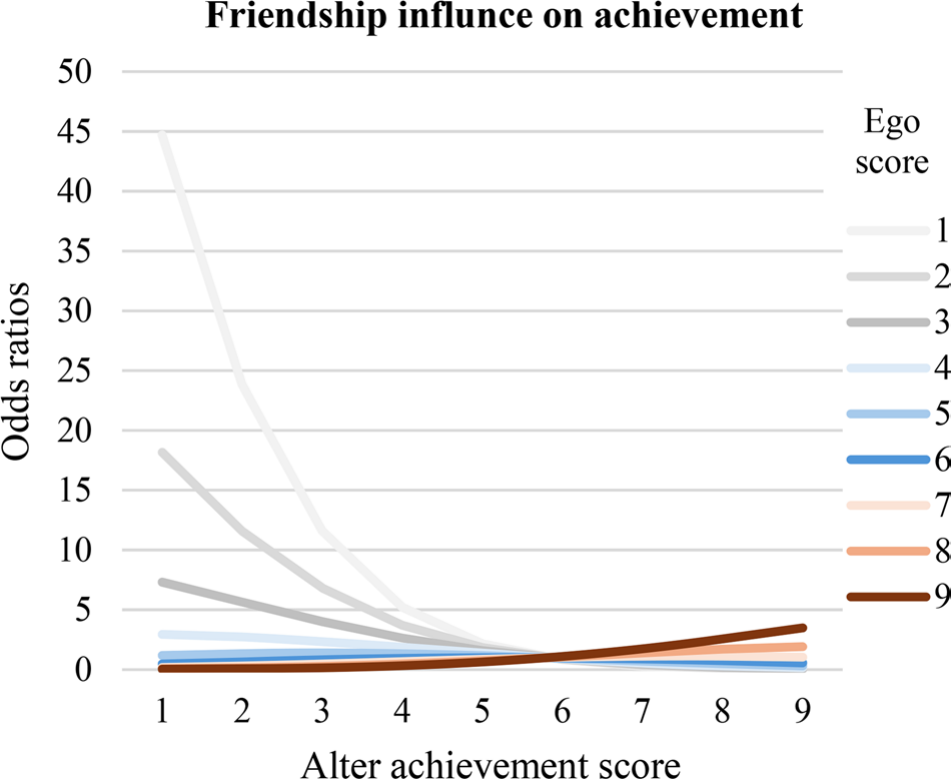
Ego-alter friendship influence regarding Chinese students’ achievement. This figure presents odds ratios quantifying the influence of a friend’s (alter’s) achievement score on a student’s (ego’s) achievement score. A score of 6 or higher is considered a passing grade. Calculations are derived from Table 3; for methodological details, see Ripley et al. (2024)

For friendship influence on achievement, Table 3 presents the results of the longitudinal SIENA analysis of friendship dynamics regarding academic achievement. Model 1 examined the simultaneous role of parental practices and friendship on academic achievement. Consistent with the Hypothesis 1, Chinese adolescents’ academic achievement was influenced to be similar to their friends’ achievement (*average alter*, *β* = 0.20, *p <* .001). The further *ego-alter* influence table analysis examined the direction of friendship influence in academic achievement. It showed that friends with lower academic achievement exerted a stronger negative influence on adolescents’ achievement than the positive influence exerted by higher-achieving friends. As shown in Fig. 1, when students (ego) scored relatively low (ranging from 1 to 6), the odds ratio decreased as friends’ (alter) scores increased, suggesting that low-achieving students were strongly influenced by their peers, especially by other low-achieving friends. Conversely, when students (ego) scored relatively high (ranging from 6 to 9), the odds ratio increased with friends’ (alter) scores increasing. While it showed some positive influence from higher-achieving friends, the effect was much weaker compared to the influence observed among low-achieving students. This suggests that peer influence was more pronounced in low-achieving groups, while high-achieving students were more independent to friendship influence.

Regarding friendship selection, adolescents’ achievement significantly predicted their friendship selection. In model 1, the *achievement alter* effect was positively significant (*β* = 0.04, *p* < .001), indicating that higher-achieving adolescents received more friendship nominations. In contrast, the *achievement ego* was negatively significant (*β* = − 0.03, *p* < .001), suggesting that adolescents with higher academic achievement were less likely to nominate others as friends. Additionally, the *achievement ego× achievement alter* was positively significant (*β* = 0.04, *p* < .001), demonstrating that adolescents were more likely to form friendships with peers who had similar levels of academic achievement. Taken together, these effects indicate that higher-achieving adolescents are more attractive but also more selective as friends, and that friendship selection processes reinforce similarity in academic achievement.

### Parental Influences, Friendship Selection and Influence, and Achievement

As for the direct effects of parental practices on academic achievement, only parental expectations significantly predicted higher achievement (*β* = 0.09, *p <* .001) in model 1, consistent with Hypothesis 2c. In contrast, parental autonomy support (*β* = 0.05, *p* = .15), behavioral control (*β* = 0.03, *p* = .27), and psychological control (*β* = − 0.04, *p* = .18) showed no significant effects on adolescents’ academic achievement, after accounting for friendship selection and influence effects.

**Fig. 2:**
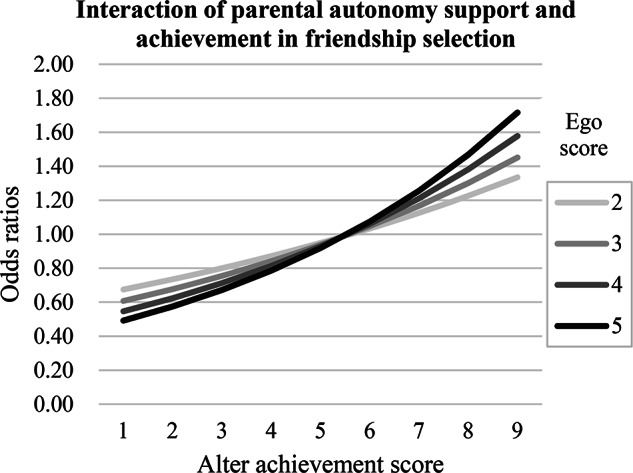
The effect of ego’s perceived parental autonomy support on the likelihood of selecting a high achieving alter as a friend. Odds ratios representing an ego’s preference for a friend (alter) based on alter’s achievement and ego’s perceived parental autonomy support. Perceived parental autonomy support was categorized by rounding original scores for clarity. A score of 6 or higher for alter’s achievement was defined as a passing grade. Analyses are based on data from Table 3 (see Ripley et al., 2024, for methodology)

As shown in Table 3, Model 2 examined the moderating role of parental practices on friendship selection and influence relating to academic achievement. Consistent with Hypothesis 3a, a significantly positive *parental autonomy support ego × achievement alter* effect was found (*β* = 0.02, *p* = .01), indicating that adolescents perceived higher levels of parental autonomy support were more likely to select friends with higher achievement. To further illustrate this pattern, *ego-alter* selection table analysis was conducted. As shown in Fig. 2, although students (egos) generally preferred friends (alters) with higher achievement, this tendency was stronger among those who perceived greater parental autonomy support. In contrast, parental expectations, psychological and behavioral control showed no significant moderating effects on friendship selection related to academic achievement.

**Table 3:** RSiena results on parental Influences, friendships selection and Influence, and achievement

	Model 1			Model 2		
	β	SE	*p*	β	SE	*p*
**Friendship selection based on Achievement**						
Achievement alter	0.04^***^	0.01	< 0.001	0.04^***^	0.01	< 0.001
Achievement ego	–0.03^***^	0.01	< 0.001	–0.03^*^	0.01	0.01
Achievement ego × Achievement alter	0.04^***^	0.00	< 0.001	0.04^***^	0.01	< 0.001
**Friendship selection: Parental influences**						
Parental autonomy support ego × achievement alter (H3a)				0.02^*^	0.01	0.01
Parental behavioral control ego × achievement alter (H3b)				0.01	0.01	0.40
Parental expectations ego × achievement alter (H3c)				0.00	0.01	0.83
Parental psychological control ego × achievement alter (H3d)				0.01	0.01	0.20
**Friendship and parental influences on achievement**						
Friends’ Achievement (average alter H1)	0.20^***^	0.03	< 0.001	0.24^***^	0.05	< 0.001
Parental autonomy support (H2a)	0.05	0.03	0.15	0.01	0.05	0.75
Parental behavioral control (H2b)	0.03	0.03	0.27	0.03	0.04	0.44
Parental expectations (H2c)	0.09^***^	0.02	< 0.001	0.08^*^	0.03	0.02
Parental psychological control (H2d)	–0.04	0.03	0.18	–0.08	0.05	0.13
**Friendship influence: Parental influences**						
Friends’ achievement × Parental autonomy support (H4a)				0.00	0.06	0.99
Friends’ achievement × Parental behavioral control (H4b)				–0.02	0.05	0.68
Friends’ achievement × Parental expectations (H4c)				0.01	0.05	0.78
Friends’ achievement × Parental psychological control (H4d)				–0.02	0.06	0.74

“Ego effect” refers to the actor who initiates friendship ties, and “alter effect” refers to the actor who receives ties. The model also included the effects of network structure, parental influences, gender, and subjective SES on friendship selection, as well as rate function, the effects of gender, subjective SES, indegree, and outdegree on achievement, see Appendix 3. 26 classes included in analysis and 20 classes converged

^*^p < .05, ^**^p < .01, ^***^p < .001

Furthermore, no significant effects of parental influences were found on friendship influence in academic achievement, which is inconsistent with Hypothesis 4. These results suggest that the strength of friends influence on adolescents’ academic achievement does not vary by their perceived parental influences.

## Discussion

Despite extensive evidence that both parents and friends influence adolescents’ academic achievement, relatively little is known about how these influences operate correctly within dynamic friendship networks. Using longitudinal social network analysis, this study simultaneously examined the effects of parental influences and friendships on adolescents’ academic achievement, as well as the moderating role of parental influences on friendship selection and influence processes related to achievement. Overall, the findings indicate that Chinese adolescents’ academic achievement is shaped both by friends and parents. Particularly, adolescents’ academic achievement became increasingly similar to that of their friends over time, with the influence of low-achieving peers being more pronounced than that of high-achieving peers. Moreover, parental expectations directly promoted higher academic achievement, while autonomy support increased adolescents’ likelihood of forming friendships with higher-achieving peers. These results underscore the roles of family and peer context in adolescents’ academic development, illustrating how parental and friendship processes collectively contribute to shaping achievement trajectories.

### How Does Friendship Influence Academic Achievement?

Consistent with Hypothesis 1, this study found that Chinese adolescents’ academic achievement became more similar to their friends’ achievement over time, even after controlling for friendship structure, friendship selection, parental factors, and demographic variables. Furthermore, results from the ego-alter influence table revealed a stronger downward influence from lower-achieving friends than an upward influence of higher-achieving friends. This pattern is consistent with studies involving Chinese adolescents (Qin et al., 2024; Shen & French, 2024) and German 5th to 7th graders (Stark et al., 2017) but contrasts with findings from Dutch (Gremmen et al., 2017), where students primarily achieve higher grades when surrounded by high-achieving friends. These findings highlight the asymmetry of peer influence based on students’ achievement levels among Chinese adolescents. Low-achieving students appear to be more vulnerable to friendship influence from other low-achieving peers. This may reflect a tendency for low-achieving students to form cohesive peer groups that reinforce similar academic behaviors and attitudes, potentially creating a cycle of underachievement. In contrast, high-achieving students appear less susceptible to the influence of their high-achieving peers. This can be attributed that high-achieving students often exhibit stronger self-regulation skills and intrinsic motivation, which may shield them from negative peer influences (Wentzel, 2017). They may also possess a greater sense of autonomy in setting and pursuing their own academic goals. Additionally, high-achieving students are already performing near the upper threshold of academic success. Thus, there is limited potential for further improvement through peer influence.

### The Direct Effects of Parental Influences on Academic Achievement

For the direct effects of parental influences on academic achievement, only parental expectations emerged as a significant predictor after accounting for friendship influences, consistent with prior evidence emphasizing their central role in adolescents’ academic performance (Pinquart & Ebeling, 2020). In contrast, autonomy support and both psychological and behavioral control showed no direct effects. This finding diverges from studies reporting significant associations between these dimensions of parenting and achievement among both Chinese adolescents (Chen et al., 2025; Zhang et al., 2024) and their Western counterparts (Pinquart, 2016).

The strong role of parental expectations aligns with expectancy–value theory (Wigfield & Eccles, 2002) and prevailing Chinese cultural norms. In Chinese families, parental expectations for academic excellence are often explicitly communicated, with academic success being closely tied to family honor and social standing (Chen et al., 2003). Parents in these contexts frequently express high academic aspirations for their children, whose internalization of these expectations as personal goals strengthens academic motivation and enhances performance. This cultural emphasis may contribute to explain why parental expectations—more than specific parenting practices—directly contribute to achievement in this sociocultural setting. This pattern is further supported by the data: correlations between parental expectations and academic achievement were relatively strong (0.40–0.43), whereas associations with autonomy support and behavioral control were modest (0.11–0.18), and negative for psychological control (–0.15 to –0.18).

Several factors may account for the nonsignificant direct effects of other parental influences. First, many previous studies identifying significant associations did not account for peer influence (Pinquart, 2016), potentially leading to inflated estimates of parental effects when friendships were uncontrolled. Second, even when significant, the effects of autonomy support and of psychological and behavioral control on achievement in previous studies tend to be modest (Jin et al., 2024; Pinquart, 2016), making their direct effects difficult to detect in models that include peer dynamics. Finally, these parental practices may affect adolescents’ long-term achievement indirectly by shaping peer experiences or moderating external socialization processes (Jin et al., 2024; Vasquez et al., 2016).

### Parenting Influences Moderating Friendship Selection and Influence on Academic Achievement

This study found that only parental autonomy support increased adolescents’ likelihood of selecting high-achieving friends. This pattern aligns with Self-Determination Theory (Ryan & Deci, 2017), which posits that autonomy-supporting parenting fosters adolescents’ independence, self-regulation, and competence. When adolescents feel empowered to make choices consistent with their personal values and goals, they are more deliberate in selecting academically oriented peers. This finding is consistent with prior research showing that autonomy-supportive parenting promotes independence in selecting friends (McCurdy et al., 2020) and the maintenance of positive peer relationships (Hu et al., 2021). In contrast, behavioral control, psychological control, and parental expectations did not significantly predict achievement-related friendship selection. This pattern can be compared with prior research showing that parental control focus typically functions to restrict or regulate adolescents’ behavior, which tends to prevent affiliation with deviant peers (Lakon et al., 2015; Soenens & Vansteenkiste, 2009) rather than promote positive friendships centered on academic pursuits. Similarly, parental expectations, although emphasizing academic achievement, may not necessarily foster the autonomy necessary for adolescents to proactively shape their social networks around achievement.

The Chinese cultural context offers a plausible explanation for this pattern. While the tendency for high-achieving students to be attractive as friends is not unique to China, the cultural salience of academic achievement in Chinese society may amplify this effect. This is reflected in distinct peer dynamics: whereas studies in Western contexts (e.g., the Netherlands) have shown mutual avoidance between high- and low-achieving adolescents (Gremmen et al., 2017), research in China suggests a more active pursuit of high-achieving peers, particularly among those with moderate grades (Qin et al., 2024). The high social value placed on academic success likely leads adolescents to internalize academic goals as personal priorities, making achievement a universally salient criterion for selecting friends irrespective of specific parental control or expectations (Chen et al., 2003). Consequently, adolescents are most able to act on this internalized goal and select more high-achieving friends when they experience autonomy and can select friends independently.

Contrary to expectations, no parental influence moderated the effects of friendship influence on academic achievement. The extent to which adolescents’ achievement became more similar to that of their friends did not differ by levels of parental influence. This finding aligns with prior research showing nonsignificant moderating role of parents’ school knowledge in buffering against the negative effects of having low-achieving friends (Im et al., 2016). One potential explanation lies in the methodological complexity of the current social network models, which simultaneously account for network structure, selection, and influence processes, thereby reducing statistical power to detect moderation effects. A more substantive explanation, however, may relate to the sociocultural environment. In China, the pervasive emphasis on educational success fosters a context in which both parental and peer influences converge around similar academic goals. This shared normative focus may produce a “ceiling effect” in academic socialization, diminishing the need for specific parental practices to moderate the already powerful peer influences towards a universally valued outcome.

### Strength, Limitations, and Future Directions

This study has several noteworthy strengths. First, the use of longitudinal social network analysis allowed for the modeling of friendship dynamics over time while accounting for friendship selection processes, thereby minimizing the risk of overestimating friendship influence. Second, the findings demonstrated both the robustness of peer effects and the unique contribution of parental expectations to adolescents’ academic achievement. Third, by testing moderation across multiple dimensions of parental influence, the study identified which parental factors most effectively supported adolescents’ friendship processes related to academic performance.

Nevertheless, this study also has some limitations. First, the study examined only a limited subset of parental factors (i.e., expectations, autonomy support, behavioral control, psychological control), excluding other dimensions such as warmth, communication, or involvement. Since parents often exhibit combined practices (e.g., being both supportive and controlling), future research should adopt a more comprehensive approach or more targeted measures of parental behaviors specifically relevant to peer dynamics to capture the multidimensional nature of parenting.

Second, the data covered only grades through 7 to 8, capturing a relatively short period during middle school. Adolescents undergo rapid developmental changes during this time. Future studies should, therefore, use longitudinal data spanning all three years of middle school to provide a deeper understanding of how these dynamics evolve over time.

Third, the study focused exclusively on within-classroom friendships, excluding cross-classroom or cross-context networks. Expanding the scope to include friendships from other classes, grades, schools, or out-of-school contexts could offer a more comprehensive understanding of peer influence.

Finally, restricting “best friend” nominations to five may have limited the full range of friendships captured. Using unlimited nominations could better represent adolescents’ friendship networks and their impact on academic outcomes (Veenstra et al., 2013). Addressing these limitations in future research will enhance understanding of the interplay between family dynamics, peer relationships, and academic achievement.

### Implications

This study advances understanding of how parenting and friendship collectively shape adolescents’ academic development and provide guidance for parents and educators seeking to enhance students’ academic outcomes. First, the positive influence of parental expectations and autonomy support suggests that parents should maintain appropriately high educational expectations while providing children with autonomy-supportive environments that foster self-regulation and the development of constructive peer relationships. Prevention and intervention programs should therefore include explicit training in autonomy-supportive parenting practice. Second, the findings reaffirm the critical importance of friendships, underscoring the needs for schools and educators to cultivate positive peer environments through collaborative learning activities, peer mentoring initiatives, and classroom structures that foster academic engagement and motivation. Finally, the study highlights the importance of tailoring interventions to different student groups. For low-achieving students, effective strategies should target the disruption of negative peer cycles by promoting positive peer dynamics and providing exposure to academic role models. For high-achieving students, individualized support, for example, including advanced learning opportunities and mentorship, is warranted, given their relative independence from peer influence.

## Conclusion

Although previous research has established the separate effects of parents and friends on academic achievement, the ways in which these influences interact within dynamic friendship networks have remained poorly understood. By integrating parental influences and friendship dynamics within a longitudinal social network framework, this study demonstrates that adolescents’ academic outcomes are shaped by both familial and peer factors. Over time, adolescents’ academic achievement converged with that of their friends, with peer influence being more pronounced among low-achieving youth. Parental expectations directly predicted academic achievement, while autonomy-supportive parenting facilitated the selection of higher-achieving friends. Together, these findings underscore the contributions of both family and peer contexts to Chinese adolescents’ academic development and delineate distinct pathways through which parents and friendships interact to shape achievement trajectories.

## Supplementary Information

Below is the link to the electronic supplementary material.

Supplementary Material 1

## Declarations

### Data Sharing Declaration

The data and code supporting the results of this study are available from the corresponding author upon reasonable request.
