# Supplementary material for: The Role of Parental Influences in Chinese Adolescents’ Academic Achievement through Shaping Friendship Network Dynamics
Source: J Youth Adolesc. 2026 Jan 5;55(5):1142–55. doi: 10.1007/s10964-025-02306-5 (PMC13156085; doi:10.1007/s10964-025-02306-5)
Supplement: Supplementary file 1 — Supplementary Material 1 [file 10964_2025_2306_MOESM1_ESM.docx]

**Online supporting information**

**The Role of Parental Influences in Chinese Adolescents’ Academic Achievement through Shaping Friendship Network Dynamics**

**Appendices**

**Table of content**

Appendix 1: Individual, friendship networks, and classroom information (Table S1 and Table S2).

Appendix 2: Parameters of the RSiena model (Table S3).

Appendix 3: The Structure and Control Parts of RSiena Results on Friendships, Achievement, and Parental Influences (Table S4).

**Appendix 1. Individual, friendship, and classroom information**

**Table S1** Overview of Individual and Classroom Information

| *Classroom number* | | 1 | 2 | 3 | 4 | 5 | 6 | 7 | 8 | 9 | 10 | 11 | 12 | 13 |
| --- | --- | --- | --- | --- | --- | --- | --- | --- | --- | --- | --- | --- | --- | --- |
| Total size^a^ | | 51 | 50 | 51 | 29 | 50 | 49 | 65 | 68 | 63 | 67 | 63 | 68 | 62 |
| Age (in years) W1 | | 12.98 | 12.84 | 13 | 12.84 | 12.98 | 12.92 | 13.36 | 12.96 | 13.17 | 12.94 | 13.28 | 13.07 | 13.19 |
| Girls | | .51 | .5 | .47 | .47 | .46 | .51 | .34 | .47 | .33 | .48 | .37 | .47 | .34 |
| *Respondents^b^* | |  |  |  |  |  |  |  |  |  |  |  |  |  |
| Present | |  |  |  |  |  |  |  |  |  |  |  |  |  |
|  | W1 | 50 | 49 | 48 | 46 | 47 | 48 | 62 | 67 | 61 | 65 | 60 | 66 | 60 |
|  | W2 | 49 | 47 | 47 | 47 | 47 | 49 | 58 | 66 | 61 | 65 | 61 | 65 | 58 |
|  | W3 | 50 | 48 | 45 | 47 | 44 | 49 | 56 | 64 | 55 | 64 | 59 | 59 | 54 |
|  | W4 | 50 | 45 | 46 | 48 | 45 | 47 | 48 | 63 | 52 | 58 | 55 | 61 | 50 |
| Missing | |  |  |  |  |  |  |  |  |  |  |  |  |  |
|  | W1 | 0 | 0 | 1 | 1 | 0 | 0 | 0 | 1 | 0 | 0 | 2 | 0 | 0 |
|  | W2 | 1 | 1 | 0 | 1 | 0 | 0 | 2 | 1 | 0 | 0 | 1 | 0 | 0 |
|  | W3 | 1 | 0 | 1 | 1 | 1 | 0 | 3 | 3 | 4 | 1 | 0 | 5 | 4 |
|  | W4 | 0 | 2 | 0 | 0 | 1 | 2 | 5 | 0 | 3 | 2 | 2 | 0 | 2 |
| Leaving | |  |  |  |  |  |  |  |  |  |  |  |  |  |
|  | W2 | 0 | 1 | 2 | 1 | 0 | 0 | 2 | 1 | 1 | 0 | 1 | 2 | 3 |
|  | W3 | 0 | 2 | 4 | 1 | 4 | 0 | 6 | 1 | 4 | 1 | 3 | 4 | 3 |
|  | W4 | 1 | 3 | 5 | 1 | 4 | 0 | 11 | 5 | 8 | 7 | 5 | 7 | 10 |
| Joining | |  |  |  |  |  |  |  |  |  |  |  |  |  |
|  | W2 | 0 | 0 | 0 | 2 | 0 | 1 | 0 | 0 | 1 | 0 | 1 | 1 | 1 |
|  | W3 | 1 | 1 | 1 | 2 | 2 | 1 | 3 | 0 | 2 | 1 | 0 | 2 | 1 |
|  | W4 | 1 | 1 | 2 | 2 | 3 | 1 | 2 | 0 | 2 | 2 | 0 | 2 | 2 |
| Staying | |  |  |  |  |  |  |  |  |  |  |  |  |  |
|  | W1-W2 | 49 | 47 | 46 | 45 | 47 | 48 | 58 | 65 | 60 | 65 | 57 | 63 | 57 |
|  | W2-W3 | 48 | 46 | 43 | 46 | 42 | 49 | 51 | 63 | 54 | 63 | 56 | 56 | 54 |
|  | W3-W4 | 49 | 45 | 43 | 47 | 44 | 47 | 45 | 60 | 49 | 56 | 53 | 56 | 45 |

Continued:

| *Classroom number* | | 14 | 15 | 16 | 17 | 18 | 19 | 20 | 21 | 22 | 23 | 24 | 25 | 26 |
| --- | --- | --- | --- | --- | --- | --- | --- | --- | --- | --- | --- | --- | --- | --- |
| Total size^a^ | | 58 | 67 | 56 | 55 | 63 | 59 | 60 | 50 | 49 | 49 | 51 | 50 | 51 |
| Age (in years) W1 | | 12.93 | 13.38 | 13.14 | 12.8 | 13.04 | 12.9 | 13.08 | 13.19 | 13 | 13.02 | 13.07 | 13 | 13.18 |
| Girls | | .62 | .37 | .68 | .64 | .44 | .63 | .4 | .44 | .45 | .45 | .45 | .46 | .49 |
| *Respondents^b^* | |  |  |  |  |  |  |  |  |  |  |  |  |  |
| Present | |  |  |  |  |  |  |  |  |  |  |  |  |  |
|  | W1 | 58 | 60 | 54 | 52 | 59 | 58 | 59 | 49 | 49 | 48 | 49 | 48 | 47 |
|  | W2 | 57 | 60 | 55 | 51 | 56 | 57 | 59 | 48 | 47 | 46 | 47 | 45 | 48 |
|  | W3 | 55 | 54 | 56 | 50 | 58 | 59 | 56 | 47 | 45 | 46 | 47 | 48 | 45 |
|  | W4 | 54 | 50 | 56 | 50 | 50 | 59 | 50 | 44 | 42 | 44 | 45 | 45 | 44 |
| Missing | |  |  |  |  |  |  |  |  |  |  |  |  |  |
|  | W1 | 0 | 4 | 2 | 0 | 0 | 0 | 1 | 0 | 0 | 0 | 0 | 0 | 0 |
|  | W2 | 0 | 2 | 0 | 0 | 2 | 1 | 1 | 1 | 2 | 2 | 1 | 1 | 0 |
|  | W3 | 0 | 4 | 0 | 0 | 1 | 0 | 3 | 0 | 2 | 1 | 0 | 0 | 1 |
|  | W4 | 0 | 0 | 0 | 1 | 7 | 0 | 4 | 2 | 1 | 0 | 0 | 2 | 1 |
| Joining | |  |  |  |  |  |  |  |  |  |  |  |  |  |
|  | W2 | 1 | 3 | 1 | 3 | 2 | 0 | 0 | 0 | 0 | 0 | 1 | 2 | 0 |
|  | W3 | 3 | 9 | 0 | 4 | 4 | 0 | 1 | 2 | 2 | 2 | 3 | 2 | 3 |
|  | W4 | 4 | 17 | 0 | 4 | 5 | 0 | 6 | 4 | 6 | 4 | 6 | 3 | 6 |
| Leaving | |  |  |  |  |  |  |  |  |  |  |  |  |  |
|  | W2 | 0 | 1 | 0 | 2 | 1 | 0 | 0 | 0 | 0 | 0 | 0 | 0 | 1 |
|  | W3 | 0 | 3 | 0 | 2 | 4 | 1 | 0 | 0 | 0 | 1 | 1 | 2 | 2 |
|  | W4 | 0 | 3 | 0 | 3 | 3 | 1 | 0 | 1 | 0 | 0 | 2 | 2 | 4 |
| Staying | |  |  |  |  |  |  |  |  |  |  |  |  |  |
|  | W1-W2 | 57 | 56 | 54 | 49 | 55 | 57 | 58 | 48 | 47 | 46 | 47 | 45 | 47 |
|  | W2-W3 | 55 | 49 | 53 | 49 | 54 | 57 | 55 | 47 | 44 | 45 | 46 | 45 | 45 |
|  | W3-W4 | 54 | 45 | 54 | 49 | 49 | 59 | 47 | 43 | 41 | 43 | 44 | 45 | 41 |

*Notes*. a, total network size over all waves, including respondents joining, leaving, and staying. b, present (missing) are those who belonged to the classroom and who fill (did not fill) out the questionnaire; Joining are those who were new in the classroom in W2 or W3 or W4; Leaving are those who left the classroom in W2 or W3 or W4; Staying are those who remained in the classroom in W1 and W2 or W2 and W3 or W3 and W4.

Table S2 Changes in Friendship Networks Variables across Two Observations in All Classrooms (*N* = 26)

| Class | Fr 0 => 0 | Fr 0 => 1 | Fr 1 => 0 | Fr 1 => 1 | Distance | Jaccard | Missing |  |
| --- | --- | --- | --- | --- | --- | --- | --- | --- |
| 1 | 2219 | 79 | 84 | 118 | 163 | .42 | .02 |  |
| 2 | 2101 | 71 | 91 | 89 | 162 | .36 | .04 |  |
| 3 | 2248 | 80 | 71 | 102 | 144 | .40 | .02 |  |
| 4 | 2048 | 69 | 74 | 113 | 143 | .44 | .02 |  |
| 5 | 2157 | 73 | 83 | 88 | 149 | .36 | .02 |  |
| 6 | 1995 | 98 | 73 | 90 | 171 | .35 | .04 |  |
| 7 | 3398 | 84 | 93 | 85 | 167 | .32 | .12 |  |
| 8 | 4007 | 108 | 126 | 114 | 224 | .33 | .04 |  |
| 9 | 3212 | 74 | 96 | 94 | 170 | .36 | .11 |  |
| 10 | 3895 | 86 | 128 | 119 | 212 | .36 | .04 |  |
| 11 | 3491 | 93 | 104 | 95 | 190 | .33 | .03 |  |
| 12 | 3900 | 109 | 113 | 110 | 214 | .33 | .07 |  |
| 13 | 3137 | 101 | 124 | 60 | 202 | .21 | .10 |  |
| 14 | 3027 | 72 | 104 | 103 | 176 | .37 | .00 |  |
| 15 | 3899 | 79 | 105 | 77 | 184 | .30 | .06 |  |
| 16 | 2783 | 91 | 76 | 130 | 155 | .44 | .00 |  |
| 17 | 3102 | 96 | 100 | 124 | 196 | .39 | .00 |  |
| 18 | 2850 | 89 | 120 | 68 | 209 | .25 | .12 |  |
| 19 | 2104 | 67 | 79 | 102 | 138 | .41 | .04 |  |
| 20 | 1982 | 65 | 66 | 95 | 131 | .42 | .06 |  |
| 21 | 2083 | 56 | 63 | 102 | 114 | .46 | .02 |  |
| 22 | 2293 | 76 | 90 | 91 | 158 | .35 | .00 |  |
| 23 | 2138 | 69 | 72 | 73 | 141 | .34 | .04 |  |
| 24 | 2220 | 69 | 89 | 72 | 151 | .31 | .04 |  |
| 25 | 3851 | 119 | 127 | 130 | 232 | .35 | .01 |  |
| 26 | 4050 | 107 | 113 | 152 | 220 | .41 | .00 |  |
| Average | 2853.46 | 83.85 | 94.77 | 99.85 | 173.69 | .36 | .04 |  |

**Appendix 2. Parameters of the RSiena** **model**

Table S3 Explanation of Some Basic Parameters in the RSiena Model

| Parameter | Effect name in Rsiena | Explanation |
| --- | --- | --- |
| *Network structures* |  |  |
| Outdegree | density | The basis tendency to form friendships |
| Reciprocity | recip | The tendency toward reciprocated friendships |
| Transitive triplets | transTrip | Tendency for a friend’ friend to become a friend |
| Transitive reciprocated triplets | transRecTrip | Tendency for friends to be more likely to become or remain mutual friends when they are part of a transitive triplet |
| Indegree popularity | inpop | Tendency for adolescents with many incoming ties to receive more ties |
| Outdegree activity | outact | Tendency for adolescents with many outgoing ties to send more ties |
| Outdegree popularity | outpop | Tendency for adolescents with many outgoing ties to receive more ties |
| *Selection effects* |  |  |
| Ego effect | egoX | The extent to which adolescents with higher levels of an attribute (e.g., achievement) send more ties |
| Alter effect^a^ | altX | The extent to which adolescents with higher levels of an attribute (e.g., achievement) receive more ties |
| Ego × Alter^b^ | egoX × altX  egoY × altX | The extent to which friendships occur more often between adolescents with a high level on an attribute (e.g., achievement)  The extent to which friendships are more likely to occur between adolescents with a high levels of attribute Y and adolescents with a high levels of attribute X |
| Same X | sameX | The tendency of adolescents to send friendship ties toward same-gender peers |
| *Influence effects* |  |  |
| Average alter^c^ | avAlt | The tendency of adolescents to become similar to their friends on a specific attribute (e.g., achievement) |
| Main covariate | effFrom | The extent to which adolescents’ own characteristics influence on own behavioral development (e.g., achievement) |
| Covariate × Average alter | avAlt × effFrom | the tendency of adolescents to become similar to their friends on a specific attribute (e.g., achievement), and whether this depends on adolescents’ own characteristics |
| Indegree | indeg | The extent to which adolescents’ incoming ties influence behaviors (e.g., achievement) |
| Outdegree | outdeg | The extent to which adolescents’ outgoing ties influence behaviors (e.g., achievement) |
| Linear shape | linear | The tendency for achievement to increase linearly over time |
| Quadratic shape | quad | The tendency for changes in achievement to depend on initial levels |

*Note*, “ego effect” refers to the actor who sends friendship ties, while “alter effect” refers to the actor who receives friendship ties. Alter effect^a^, Ego × Alter^b^, and Average alter effect^c^ are the effects for testing our hypothesis, and the others act as control variables. All of these effects are about “eval”, which examines the presence of ties regardless of whether they are newly created or maintained in the selection part and does not distinguish between upward and downward changes in the behavioral influence part.

**Appendix 3: The Structure and Control Parts of RSiena Results on Friendships, Achievement, and Parental Influences**

Table S4 the Structure and Control Parts of RSiena Results on Friendships, Achievement, and Parental Influences

|  | | Model 1 | |  | Model 2 |  |  |
| --- | --- | --- | --- | --- | --- | --- | --- |
|  | *β* | SE | *p* |  | *β* | SE | *p* |
| **Network dynamics: Friendships** |  |  |  |  |  |  |  |
| Rate period 1 | 8.70^***^ | 0.27 | <.001 |  | 8.64^***^ | 0.30 | <.001 |
| Rate period 2 | 7.77^***^ | 0.23 | <.001 |  | 7.91^***^ | 0.26 | <.001 |
| Rate period 3 | 6.83^***^ | 0.26 | <.001 |  | 6.96^***^ | 0.24 | <.001 |
| Outdegree (density) | –1.05^***^ | 0.11 | <.001 |  | –1.15^***^ | 0.11 | <.001 |
| Reciprocity | 1.64^***^ | 0.03 | <.001 |  | 1.65^***^ | 0.04 | <.001 |
| Transitive triplets | 0.54^***^ | 0.02 | <.001 |  | 0.54^***^ | 0.02 | <.001 |
| Transitive recipr. triplets | –0.21^***^ | 0.02 | <.001 |  | –0.22^***^ | 0.03 | <.001 |
| Indegree - popularity | 0.04^***^ | 0.01 | <.001 |  | 0.04^***^ | 0.01 | <.001 |
| Outdegree - popularity | –0.23^***^ | 0.02 | <.001 |  | –0.23^***^ | 0.02 | <.001 |
| Outdegree - activity | –0.18^***^ | 0.01 | <.001 |  | –0.18^***^ | 0.01 | <.001 |
| **Friendship selection: Parental influences** |  |  |  |  |  |  |  |
| Parental expectations alter | 0.03^*^ | 0.01 | .01 |  | 0.03^*^ | 0.01 | .02 |
| Parental expectations ego | 0.01 | 0.01 | .54 |  | 0.01 | 0.01 | .35 |
| Parental expectations ego × Parental expectations alter | 0.00 | 0.01 | .92 |  | 0.00 | 0.01 | .89 |
| Parental psychological control alter | 0.01 | 0.01 | .49 |  | 0.00 | 0.02 | .89 |
| Parental psychological control ego | –0.01 | 0.02 | .43 |  | –0.01 | 0.02 | .48 |
| Parental psychological control ego × Parental psychological control alter | –0.03^*^ | 0.02 | .02 |  | –0.04^*^ | 0.02 | .04 |
| Parental behavioral control alter | 0.01 | 0.01 | .35 |  | 0.02 | 0.01 | .17 |
| Parental behavioral control ego | 0.03 | 0.03 | .24 |  | 0.01 | 0.02 | .75 |
| Parental behavioral control ego × Parental behavioral control alter | 0.01 | 0.01 | .26 |  | 0.01 | 0.01 | .40 |
| Parental autonomy support alter | 0.02 | 0.01 | .07 |  | 0.03^*^ | 0.01 | .03 |
| Parental autonomy support ego | 0.02 | 0.02 | .25 |  | 0.01 | 0.02 | .70 |
| Parental autonomy support ego × Parental autonomy support alter | 0.03^*^ | 0.01 | .01 |  | 0.04^*^ | 0.01 | .01 |
| **Friendship selection: Controls** | |  |  |  |  |  |  |
| subSES alter | –0.02 | 0.01 | .09 |  | –0.03 | 0.02 | .09 |
| subSES ego | 0.00 | 0.02 | .94 |  | –0.01 | 0.02 | .65 |
| subSES ego x subSES alter | 0.03 | 0.02 | .18 |  | 0.02 | 0.02 | .37 |
| Gender alter | 0.07 | 0.04 | .07 |  | 0.06 | 0.04 | .17 |
| Gender ego | –0.16^*^ | 0.06 | .01 |  | –0.14^*^ | 0.07 | .03 |
| Same Gender | 0.94^***^ | 0.05 | <.001 |  | 0.95^***^ | 0.05 | <.001 |
| **Network dynamics: Achievement** | |  |  |  |  |  |  |
| Rate period 1 | 0.94^***^ | 0.06 | <.001 |  | 0.92^***^ | 0.06 | <.001 |
| Rate period 2 | 1.70^***^ | 0.13 | <.001 |  | 1.70^***^ | 0.18 | <.001 |
| Rate period 3 | 1.23^***^ | 0.10 | <.001 |  | 1.20^***^ | 0.10 | <.001 |
| achievement linear shape | –0.06 | 0.11 | .60 |  | –0.05 | 0.14 | .74 |
| achievement quadratic shape | –0.07^***^ | 0.01 | <.001 |  | –0.07^***^ | 0.02 | <.001 |
| achievement indegree | 0.02 | 0.02 | .20 |  | 0.03 | 0.02 | .28 |
| achievement outdegree | 0.04 | 0.04 | .36 |  | 0.02 | 0.05 | .71 |
| subSES | 0.06 | 0.03 | .06 |  | 0.03 | 0.04 | .40 |
| Gender | 0.10^*^ | 0.04 | .02 |  | 0.10 | 0.06 | .10 |

*Note*. Indegree – popularity and outdegree – popularity represent the dynamics of in and out degrees.

^*^*p* <.05, ^**^*p* <.01, ^***^*p* <.001.

As shown in Table S4, Network structural effects were similar in both model 1 and model 2. The results of model 1 will be reported. A negative effect for density indicated that youth were selective in their friendships [*β* = –1.05, *p* < .001]. Moreover, youth reciprocated friendships [*β* = 1.64, *p* < .001] and were likely to become friends with their friends’ friends [*β* = 0.54, *p* < .001]. Adolescents who received many nominations received more nominations over time [*β* = 0.04, *p* < .001]. Adolescents who gave many nominations decreased in nominations received over time [*β* = –0.23, *p* < .001], and gave fewer nominations over time [*β* = –0.18, *p < .001*]. Youth selected same-gender peers as friends [*β* = 0.94, *p* < .001], and girls tended to send fewer friendship nominations than boys [*β* = -0.16, *p* < .001].

In model 1, we also included the effects that how parental influences and adolescents’ achievement shape adolescents’ friendship selection. Parental expectations and parental behavioral control were found to have no significant effects on adolescents’ friendship selection. This was consistent across *ego, alter*, and *ego×alter* effects. The *ego×alter* effect for parental autonomy support was positively significant (*β* = 0.04, *p* = .01), indicating that adolescents with higher levels of parental autonomy support tend to select friends with similar levels of parental autonomy support. Furthermore, the *ego×alter* effect for parental psychological control was negatively significant (*β* = –0.04, *p* = .04), suggesting that adolescents with higher levels of parental psychological control tend to select friends with dissimilar levels of parental psychological control.

Regarding behavior dynamics, the significant and negative *quadratic shape* parameter suggests that changes in academic achievement are inverted U-shaped [*β* = –0.07, *p < .01*]. Adolescents who nominated more peers as friends (outdegree) or were being nominated as friends (indegree) had no significant effects on adolescents’ academic achievement. Girls had higher academic achievement than boys [*β* = 0.10, *p < .0*5].
